# Beyond cost-effectiveness, morbidity and mortality: a comprehensive evaluation of priority setting for HIV programming in Uganda

**DOI:** 10.1186/s12889-019-6690-8

**Published:** 2019-04-01

**Authors:** Lydia Kapiriri, Na-Mee Lee, Lauren Jean Wallace, Brendan Kwesiga

**Affiliations:** 1 Department of Health, Aging and Society KTH-236, McMaster University, Main Street West, Hamilton, ON 1280 Canada; 2 Global Health program, McMaster University, Hamilton, ON Canada; 3 Department of Health, Aging and Society, McMaster University, Hamilton, ON Canada; 4 World Health Organisation, Uganda Country Office, Kampala, Uganda

**Keywords:** Priority setting, Evaluation, Evaluation framework, HIV, Low income countries, Uganda

## Abstract

**Background:**

While there has been progress in controlling the HIV epidemic, HIV still remains a disease of global concern. Some of the progress has been attributed to increased public awareness and uptake of public health interventions, as well as increased access to anti- retroviral treatment and the prevention of vertical HIV transmission. These interventions would not have been possible without substantial investments in HIV programs. However, donor fatigue introduces the need for low income countries to maximize the benefits of the available resources. This necessitates identification of priorities that should be funded. Evaluating prioritization processes would enable decision makers to assess the effectiveness of their processes, thereby designing intervention strategies. To date most evaluations have focused on cost-benefit analyses, which overlooks additional critical impacts of priority setting decisions. Kapiriri & Martin (2010) developed and validated a comprehensive framework for evaluating PS in low income countries.

The objective of this paper report findings from a comprehensive evaluation of priority setting for HIV in Uganda, using the framework; and to identify lessons of good practice and areas for improvement.

**Methods:**

This was a qualitative study based on forty interviews with decision makers and policy document review. Data were analysed using INVIVO 10, and based on the parameters in Kapiriri et al’s evaluation framework.

**Results:**

We found that HIV enjoys political support, which contributes to the availability of resources, strong planning institutions, and participatory prioritization process based on some criteria. Some of the identified limitations included; undue donor and political influence, priorities not being publicized, and lack of mechanisms for appealing the decisions. HIV prioritization had both positive and negative impacts on the health system.

**Conclusions:**

The framework facilitated a more comprehensive evaluation of HIV priority setting. While there were successful areas, the process could be strengthened by minimizing undue influence of external actors, and support the legitimate institutions to set priorities and implement them. These should also institute mechanisms for publicizing the decisions, appeals and increased accountability. While this paper looked at HIV, the framework is flexible enough to be used in evaluating priority setting for other health programs within similar context.

**Electronic supplementary material:**

The online version of this article (10.1186/s12889-019-6690-8) contains supplementary material, which is available to authorized users.

## Background

HIV/AIDS has historically been, and continues to be, a highly prioritized health issue since the declaration of an epidemic in the 1980’s. The inclusion of HIV/AIDS in the Millennium Development Goals (MDGs) in 2000 resulted in increased funding and assistance from various multilateral, bilateral, public, and private agencies [1]. More funds are allocated by development assistance partners (DAPs) to HIV/AIDS than any other singular health issue. For example, in 2014, $9.71 billion USD was allocated for the disease alone, accounting for 27% of all foreign development assistance ($35.98 billion USD) [2]. In some sub-Saharan African countries, HIV funding from DAPs was found to be either equal to or greater than the host country’s budget for its entire health sector [3]. For example in Uganda, funding for HIV/AIDS is thought to have made up more than a quarter of the government’s total budget for health [4]. However, this trend may be changing.

Possibly due to the global economic down turn and donor fatigue, there have been noticeable reductions in support for health programs in low income countries [5, 6]. This is critical to programs such as HIV/AIDS which have enjoyed high donor support. Within this context of reduced resources, countries must aim to ensure that priority setting and resource allocation for HIV/AIDs is strengthened in order to make the best use of available resources.

There is a growing body of literature on priority setting for HIV. For example, Tromp et al., (Indonesia) [7, 8]; Cleary, et al. (South Africa) [9]; Kabaniha (Uganda) [10] identify the need for national HIV priority setting institutions to use explicit frameworks such as Accountability for Reasonableness (A4R), equity-efficiency mathematical modelling, multi-criteria decision analysis (MDCA), and Assessing Cost-Effectiveness (ACE). Another body of literature discusses the role of different stakeholders and criteria in priority setting. This literature reports on the influence of development assistance partners’ (DAPs) priorities on national level priority setting [11]. Other studies have revealed inconsistencies in the criteria and principles used to guide the prioritization processes, while others highlight the need for strong institutions with the capacity and leadership to implement credible priority setting processes [12, 13]. However, limited literature focuses on comprehensive evaluation including aspects of the politics of priority setting.

Priority setting, if done well, could contribute to reducing inequities in health and health outcomes [10, 14, 15]. Comprehensive evaluation would contribute to our understanding of the enablers and barriers to successful priority setting, and enable us to identify areas where improvements are necessary. This paper uses a validated framework to provide a comprehensive evaluation of priority setting for HIV in Uganda in order to identify key areas for improvement.

The specific objectives of the paper are:

1. To describe and evaluate national level priority setting and resource allocation processes for HIV in Uganda using an internationally validated framework for evaluating priority setting in low income countries.
2. To identify the enablers and barriers to HIV prioritization in Uganda and the lessons of good practice that can be shared within similar contexts.
3. To discuss the degree to which the evaluation framework was robust enough for evaluating priority setting for HIV in Uganda.

## Methods

### The conceptual framework

Kapiriri & Martin (2010) developed a comprehensive framework for evaluating priority setting (PS) in low income countries [16]. The framework is based on the literature on priority setting in low income countries (LICs) and interviews with experts in PS. While it draws upon the literature on priority setting in high income countries, it also recognizes the unique contexts of PS in LICs. The framework was validate by researchers and global health policy makers and revised [17]. The validated framework comprises of five domains, namely: (i) The PS context, (ii) The pre-requisites, (iii) The prioritization process, (iv) implementation, and (v) outcome and impact. Each domain has a number of parameters and each parameter has objectively verifiable indicators (OVI) and means of their verification (MOV). These are summarized in Table 1.

**Table 1:** Parameters for evaluating priority setting with corresponding means of verification and indicators

Parameters of Successful Priority Setting	Objectively Verifiable Indicators (OVI)	Means of Verification (MOV)
Contextual Factors		
Conducive political, economic, social and cultural context	Relevant contextual factors that may impact priority setting	Follow up intermittent interviews with local stakeholders, systematic longitudinal observations, relevant reports, media
Pre-requisites		
Political will	Degree to which politicians support the set priorities	Follow up intermittent interviews with local stakeholders, systematic longitudinal observations, relevant reports, media
*Resources*	Budgetary and human resource allocation to the health sector	National budget documents
*Legitimate and credible priority-setting institutions*	Degree to which the priority setting institution can set priorities; public confidence in the institution	Stakeholder and public interviews
Incentives	Material and financial incentives	National budget documents
The Priority Setting Process		
Stakeholder participation	Number of stakeholders participating, number of opportunities each stakeholder expresses opinion	Observations/minutes at meetings, media reports, special reports
Use of clear priority setting process/tool/methods	Documented priority setting process and/or use of priority setting framework	Observation/minutes at meetings, media reports, special reports
Use of explicit relevant priority setting criteria	Documented/articulated criteria	Observations/minutes at meetings, media reports, special reports
*Use of evidence*	Number of times available data is resourced/number of studies commissioned/strategies to collect relevant data	Observations/minutes at meetings, media reports, special reports
*Reflection of public values* Publicity of priorities and criteria Functional mechanisms for appealing the decisions	Number and type of members from the general public represented, how they are selected, number of times they get to express their opinion, proportion of decisions reflecting public values, documented strategy to enlist public values, number of studies commissioned to elicit public values Number of times decisions and rationales appear in public documents Number of decisions appealed, number of decisions revised	Observations/minutes at meetings, study reports, meeting minutes and strategic plans Media reports Observations/minutes at meetings, media reports, special reports
Functional mechanisms for enforcement	Number of cases of failure to adhere to priority-setting process reported	Observations/minutes at meetings, media reports, special reports
Efficiency of the priority-setting process	Proportion of meeting time spent on priority setting, number of decisions made on time	Observations/minutes at meetings, annual budget documents, health system reports
Implementation		
Decreased dissentions	Number of complaints from stakeholders	Meeting minutes, media reports
*Allocation of resources according to priorities*	Degree of alignment of resource allocation and agreed upon priorities, times budget is re-allocated from less prioritized to high prioritized areas, stakeholder satisfaction with decisions	Annual budget reports, evaluation documents
Decreased resource wastage	Proportion of budget unused, drug stock-outs	Budget documents, evaluation reports
Increased stakeholder understanding, satisfaction and compliance with the priority setting process	Number of stakeholders attending meetings, number of complaints from stakeholders, % stakeholders that can articulate the concepts used in priority setting and appreciate the need for priority setting	Observations/minutes at meetings, special reports, SH satisfaction survey, media reports, stakeholder interviews, evaluation reports
*Improved internal accountability/reduced corruption*	Number of publicized resource allocation decisions	Evaluation reports, stakeholder interviews, media reports
Strengthening of the priority setting institution	Indicators of increased efficiency, use of data, quality of decisions, appropriate resource allocation, % stakeholders with the capacity to set priorities	Training reports, evaluation reports, budget documents
Impact on institutional goals and objectives	% of institutional objectives met that are attributed to the priority setting process	Evaluation reports, special studies
Outcome/Impact		
*Impact on health policy and practice*	Changes in health policy to reflect identified priorities	Policy documents
*Achievement of health system goals*	% reduction in DALYs, % reduction of the gap between the lower and upper quintiles, % of poor populations spending more than 50% of their income on health care, % users who report satisfaction with the healthcare system	Ministry of Health documents, Demographic and Health Surveys, commissioned studies
*Improved financial and political accountability*	Number of publicized financial resource allocation decisions, number of corruption instances reported, % of the public reporting satisfaction with the process	Reports, media reports, interviews with stakeholders
Increased investment in the health sector and strengthening of the health care system	Proportion increase in the health budget, proportion increase in the retention of health workers, % of the public reporting satisfaction with the health care system	National budget allocation documents, human resources survey reports, interviews with stakeholders, media reports

*Italics: Parameters related to the health system strengthening*; Source: (Kapiriri, 2017)

### Data collection

This was a qualitative study based on key informant interviews and a review of relevant policy documents and analysis of media reports published regarding the PS process for HIV.

Setting: The study was conducted at the national and district levels in Uganda (2014–2016).

#### Interviews

The interviews were conducted by three trained Ugandan research assistants and LK who interviewed forty respondents were interviewed from the national and district levels (Table 2). Respondents were identified by virtue of their knowledge of the prioritization processes for HIV. National level respondents included respondents from multilateral and bilateral DAPs, national and local government, researchers and Non-Governmental Organizations.

**Table 2:** Respondents by level of decision making

Respondent Type	Number of Respondents
*National development Agencies*	13
*National Government*	11
*District Government*	16
*Total*	40

*Sampling*: Snow ball sampling was used to identify the respondents. The index respondent was the national lead for HIV programs within the ministry of health. This respondent identified subsequent respondents, who in turn identified additional respondents they deemed relevant to the study objectives.

#### Data collection

The interviews were conducted by trained research assistants using a pilot tested interview guide (Additional file 1). The questions were based on the parameters in the evaluation framework. However, the interview guide was used with flexibility to allow the interviewer to pursue any emerging themes. Respondents were asked about the most recent HIV strategic planning and prioritization (2010–2015).

#### Document review

BK obtained and reviewed policy documents as well as grey literature from key institutional websites of the Ministry of Health (MOH), Uganda AIDS Commission (UAC), The AIDS Support Organization (TASO) and the Ministry of Gender, Labor and Social Development.

Relevant documents included the health sector strategic plans [18–20], HIV specific plans [21–23], and the HIV prevention strategy [24]. Information related to priority setting and the parameters identified in the evaluation framework was abstracted and summarized to supplement the interview findings.

Media reports covering the period 2010–2015 from the two main Ugandan daily newspapers, the Daily Monitor and New Vision, were also reviewed. The search engines LexisNexis and Factiva were used. A structured search strategy was developed using the terms HIV and AIDS combined with common key terms or phrases from HIV-related policies, interventions and populations. Articles containing relevant information related to the domains and parameters identified in evaluation framework [16, 17] were summarized in a review. A total of 1173 articles were generated and 418 were included in the review. Information relevant to each parameter was summarized and then triangulated with information from the document review and interviews and representative media reports illustrative of key findings were selected.

### Data analysis

Interview data were analyzed using NVIVO 10. Consistent with qualitative data analysis, the focus of the analysis was on identifying common themes across the interviews as opposed to reporting individual responses [24]. To facilitate this, the initial step was to identify the initial codes, three researchers independently coded three interviews, creating a code book. They met and discussed the codes and resolved any discrepancies. Then one individual used the agreed upon codes to code and analyze the rest of the interviews. Related codes were grouped together under larger categories, and in turn, related categories were grouped under themes [24]. Secondary analysis included comparing the derived themes to the parameters within the framework and assessing the degree to which the identified themes conformed to the parameters.

The results section is organized according to these parameters, reporting the convergence of most of the responses for each parameter, and where present, any divergences are also reported.

## Results

Forty respondents discussed issues related to priority setting for HIV at the national level and were included in the data analysis. Of these, thirteen worked with international HIV/AIDS programs, and the rest worked with the Uganda Ministry of Health, either with the Uganda AIDS commission or in other programs but were knowledgeable of priority setting for HIV/AIDS, although they did not directly work with HIV/AIDS programs. The respondents from the Districts comprised of members of the District Health Teams. Consistent with qualitative methodologies, their individual responses were aggregated and emerging themes identified [24]. Emerging themes from the interviews, as well as the findings from the document and media review are organized according to the broad themes labeled according to the five domains within the framework namely; the priority setting context, the pre-requisites, the PS process, implementation, impact and outcome. For anonymity, respondents were allocated to signify their organizations and the level at which they work: N = Government, national level respondent; D = Government, district level respondents; DAP = development assistance partner, NGO = Non-governmental organization. The results are summarized in Table 3.

**Table 3:** Evaluating priority setting for HIV in Uganda using the parameters of successful priority setting

Parameters of Successful Priority Setting	HIV Case Study
Contextual factors	
Conducive political, economic, social, cultural context	*Political:* Political stability positively impacted priority setting and implementation. *Economic:* Disagreements between DAPs and Ugandan government (homosexuality bill) led funds to be reduced temporarily, this impacted implementation, for instance, by reducing the availability of ARVs. Global contraction in funds impacted the health sector and HIV programs *Sociocultural:* Disagreements between DAPs and Ugandan government over the Homosexuality Act reduced funds for implementation temporarily. Cultural and religious beliefs posed implementation challenges for priorities such as family planning and male circumcision; low education levels and stigma and discrimination were barriers to prevention and treatment *Legal:* Prevention and Control Act increased discrimination and stigma
Prerequisites	
Political will	Strong political commitment from key politicians such as the President.
Resources	Small MOH budget for health; decreased level of funding from DAPs was also observed, although overall, they continued to invest large amounts of funding for HIV.
Legitimate and credible priority-setting institutions	UAC has technical expertise and their political appointment, however, their role is sometimes undermined (UAC, 2016).
Incentives	None discussed
Prioritization process	
Stakeholder participation	PS is participatory and involves representatives from the districts, CSOs, FBOs, DAPs, politicians and the private sector; DAPs sometimes negatively influence the agenda; CSOs, as community representatives lack capacity to participate
Use of clear priority setting process/tool/method	None reported aside from BOD/CEA
Use of explicit/relevant priority setting criteria	Epidemiological evidence, cost-effectiveness, local context, resource availability, alignment with national priorities, alignment with international declarations, accountability, politics, equity, and value added.
Use of evidence	Epidemiological evidence, evidence of cost effectiveness, beneficiary assessment data
Reflection of public values	The public was directly involved via consultations and annual district partnership meetings
Publicity of priorities and criteria	Some government priorities were discussed. Rationales for prioritization inconsistently discussed
Functional mechanisms for appealing the decisions	None reported. Complaints channeled through the media
Functional mechanisms for enforcement	None reported
Efficiency of the priority setting process	PS process reportedly efficient; delays in implementation including slowness in procuring and releasing funds
Implementation	
Allocation of resources according to priorities	More resources allocated to curative care rather than prevention. Majority of prevention budget funded externally, however donor funds may have negatively impacted the implementation of national priorities.
Decreased resource wastage	Expiry of ARVs in clinics and national medical stores
Improved internal accountability/reduced corruption	Off-budget system makes funding difficult to track; two cases of possible corruption – the Global Fund and OPM scandals – were reported
Increased stakeholder understanding, satisfaction and compliance with the priority setting process	Satisfaction with identified prevention priorities and level of external funding, however, sense that DAPs did not comply with national priorities, as in the case of circumcision
Decreased dissentions	Dissentions over the government’s prioritization of treatment while inadequately focusing on prevention strategies and the negative impact of the HIV Prevention and Control Bill. Some members of the public disagreed with male circumcision as a prevention strategy
Impact and Outcomes	
Strengthening of the priority setting institution	ACP and UAC may have been strengthened in terms of capacity and financial resources
Impact on PS Institution goals and objectives	Goals of ACP and UAC to contribute to HIV control were achieved, to some extent
Impact on Health Policy and Practice	Some policy changes, such as the prioritization of male circumcision as a preventative strategy occurred
Achievement of Health System Goals	*Improvement of population health:* Notable improvements, including declining transmission and increased access to ARVs *Fairness in financial contribution:* Although public funding for the HIV response increased during the period, out of pocket contributions were still high, especially for vulnerable groups *Responding to the public’s expectations:* Not reported
Improved financial and political accountability	Deliberate reduction in funding from donors due to issues with poor financial accountability in 2006 and 2012, as discussed above.
Increased investment in the health sector and strengthening of the health care system	Despite increases in funding, gaps in funding, as well as concerns about sustainability and predictability of donor spending and the impact of vertical funding on the health system were reported

### The priority setting context

The evaluation framework recognizes that an evaluation of a PS process is incomplete without taking into account the economic, political, and sociocultural context in which priorities are identified and implemented [16, 17].

The reviewed documents mentioned that all strategies were based on the assumptions that there would be continued economic stability and growth, political stability and leadership, accountable and transparent financial management, increased health budget allocation, and continued financial support from DAPs [20, 24]. Some of the interviews, however, revealed that the context in which the priorities were set during the time of study may have not been conducive to successful prioritization and implementation, as discussed below.

#### Economic context

According to the reviewed documents, the reduction in donor funding impacted the general availability of resources for the health sector, as well as HIV programming. However, they still reported that HIV continues to enjoy disproportionate donor support, notably through programs such as the Global Fund, Presidents Emergency Program for AIDs Relief.

#### Political context

During the study period, there seemed to have been political stability within the country. However, the interviews revealed that the implementation of priorities may have been impeded due to cultural and political disagreements between the DAPs and the Ugandan government regarding the Uganda Anti-Homosexuality Act. This is also thought to have contributed to the economic context discussed above.

*“…we do this work in collaboration, our partners supporting us, but of late we have had issues with some of those partners who are withdrawing for example the Americans. When the President signed their Anti-Homosexuality Act they went on a slow down.” (N_1).*

This sentiment was exemplified by the patterns in donor funding for HIV over this period, whereby a somewhat downward trend was observed [25]. (Table 4).

**Table 4:** Trends in percentage HIV/AIDS funding by source [25]

	2011/12	2012/13	2013/14	2014/15
Private	0^a^	0	0	1
Other Donors	7	7	6	6
Multi-lateral donors	5	5	4	3
Global fund	10	10	13	19
Bilateral donors	4	5	4	4
PEPFAR	61	60	59	55
Public	13	14	14	12
Total funding in US$ millions	425	411	470	551

^a^percentages

#### Sociocultural context

Cultural beliefs are thought to have hindered implementation of the priorities. The reviewed media articles highlighted the influence of religious doctrine on communities’ and religious leaders’ acceptance of interventions such as condoms, media reports also described how the larger sociocultural context, including power differences between married couples [26, 27], low education levels and lack of HIV knowledge in rural communities [28, 29], as well as stigma and discrimination against HIV positive people [30, 31] were barriers to HIV prevention and treatment. The media reports collaborated with the findings from the interviews, where respondents discussed the influence of culture on the acceptance of different interventions such as circumcision;

*“…Then of course sometimes we get political, religious interests and one of those of course is ABC. There have been mixed messages. You saw in a paper recently women in the north have rejected men who are circumcised. So there are other cultural biases that sometimes make it difficult…” (N_20).*

#### Legal environment

The reviewed documents and the interviews identified two acts that were passed during the study period that may have impacted priority setting and implementation of HIV interventions. The prevention and control act, which required disclosure, was thought to have increased discrimination and stigma—and may have impacted people’s willingness to be screened. The 2014 Anti-homosexuality Act, although later repealed, had repercussions for DAP funding, as discussed above. Concurrent with these, guidelines for a collective bargaining act for employee’s negotiation for better terms with regards to non-discrimination of HIV positive employees were published [32].

### Prerequisites

According to the evaluation framework, prerequisites (factors that need to be in place in order for PS to succeed) include: political will, or government commitment to setting and implementing priorities, the availability of resources necessary to support PS, the credibility and capacity of the institution carrying out PS activities, and the presence of incentives to ensure adherence to the identified processes and priorities [16, 17]. We discuss these in detail below.

#### Political will

The history of HIV/AIDS efforts in Uganda demonstrates the importance of political will for successful prioritization. From its onset, HIV/AIDS has enjoyed high levels of support from the President, as reported in the media [33] and in the reviewed documents [24]. Furthermore, the First Lady’s is reportedly a champion for the expansion of the expanded maternal to child transmission (eMTCT) program [24, 34]. These findings were corroborated with the interviews as expressed by one respondent;

*“…The President I think you may have heard he has been known as a champion for the HIV/ AIDS program, he’s re-known worldwide for that. He has gone out right from a long time ago, up to now he still is very supportive…” (N_1).*

#### Resources

While HIV programs have enjoyed national and international support and has relatively more funding relative to other health programs, the interviews revealed that inadequate financial and human resources (HR) served as a challenge to successful prioritization. A decreased level of funding from DAPs was observed, although overall, they continued to invest large amounts of funding for HIV. During the period of the NSP I, 2007/2008–2011/2012, there was an increase in the budgetary allocation for HIV/AIDS from US$14 M to 53 M and an estimated increase to $70 M in 2014/2015 [34, 4]. Though as a share of the government’s entire current health expenditure, HIV/AIDS funding decreased from 36% in 2010/2011 to 32% in 2013/2014 [19, 20]. Furthermore, while the implementation of the NSP for 2015/16 was projected to require US$ 3647 million (of which 55% was allocated to Care and treatment, 23% allocated to prevention, 4% to Social support, and 18% allocated to system strengthening); only USD$2668 million were projected to be available—leaving a financial gap of USD$918 million by 2019/20. [25]. The reduction in funding was reported to have contributed to implementation challenges, as discussed by a respondent:

*“…Funding challenges constrict the number of things that can be done by DAPs. I would say that the only one is that we’d like to do more but we can’t because we face challenges of funding challenges…So I…I don’t think we have really challenges other than the constraints that we have in meeting all the needs that we meet in the districts or the implementing sites, yeah…” (DAP_ 1).*

reports also reflected on the high contribution of donor funding to Anti- retroviral treatment [35, 36]. They also commented on the consequences of the conflict between donor and national policies; for example when the government passed the homosexuality bill, and there were retribution cuts by the Global Fund for Anti-retroviral treatment and faith-based HIV programs [37, 38].

#### Legitimate and credible PS institutions

The AIDs Control Programme (ACP) and Uganda AIDs Commission (UAC) are the key priority setting institutions for HIV/AIDS in Uganda. The UAC is a separate entity from the MOH and is housed under the Office of the President. The UAC was put in charge of overseeing the development and implementation of HIV/AIDS policy and coordination of all HIV related programs in Uganda [20, 22, 23, 34]. The legitimacy of the UAC is by virtue of their technical expertise and their political appointment. However, the reviewed documents reveal that role of the UAC is sometimes undermined because of the numerous players’ limited understanding of the existing structures [34].

#### Incentives

No incentives were discussed in the reviewed documents or by respondents. Although one would allude to the availability of resources as an incentive for the health workers and those working with the HIV programs.

### The priority setting process

The framework identifies nine parameters under this domain. We discuss these in detail.

#### Stakeholder participation

The documents and interviews revealed that the HIV/AIDS priority setting is participatory. Stakeholder participation is in the form of consultations, for example, through the Joint Annual Review of the Uganda HIV/AIDS Partnership; District Consultations that are organized in eight regional groups and attended by stakeholders from all the districts; DAP meetings, and meetings with the Sector Ministries, civil society organizations, and the Parliamentary Sub-Committees on HIV/AIDS, the president, and the private sector. The Ministry of Health Officers take the lead in providing technical guidance as well as resources for implementing HIV activities. The DAPs provide funding and technical support, while district officers, community representatives, civil society organizations, and faith-based organizations were thought to represent the views of the public. While people living with HIV were thought to be involved in the process, there was emphasis on the need to ensure that their needs are reflected;

*“…I think the, although we say national level the stakeholders should be the community. One of the most important stakeholders should be the community. What are their needs? There should be a deliberate effort to get them to input into the planning and budget allocation process. So that one should be the most important stakeholder…” (NGO_4).*

The role of DAPs and politicians was legitimized by some respondents and documents. For example WHO was recognized for its role in providing evidence based standards:

*“…One of the roles of WHO is setting norms and standards for health care. For HIV … to reach those norms WHO does a lot of research, reviews existing studies on this subject (to develop generic recommendations which are translated to different countries to choose out what the country thinks are the priorities within this generic guidance…” (DAP_3).*

However, there were perceptions that sometimes DAPs may introduce their agendas in the process. There was also concern that the public, and civil society organizations who should be involved in the process are limited by their lack of capacity for meaningful.

*“…but also my experience is that civil society which is supposed to be engaging on the part of the community does not have the capacity to do analysis and budgets and put up an argument because Ministry of Health will say ok if you don’t like this which one do you like? Go and give us your own perspective on paper of what you think needs to happen. And sometimes for civil society this is a challenge…” (NGO_5).*

#### Use of clear priority setting process/tools/methods

Neither the documents and policy reports nor the interviews indicated that explicit priority setting tools or methodologies were used, aside from the Burden of disease and cost- effectiveness analysis which was used in the development of the national essential healthcare package.

#### Use of explicit relevant priority setting criteria

The documents and respondents identified several factors that may have informed the identification of HIV priorities. These included: epidemiological evidence on the prevalence and mortality rates of HIV, cost-effectiveness of the interventions, accountability, politics, equity, value added, feasibility (including; local context, resource availability), alignment with national priorities, alignment with international declarations. Cost effectiveness, was identified as a critical criterion, which they claimed to have been used to justify increasing the number of people receiving anti-retroviral treatment. While equity was given as one of the factors that influenced the decision to prioritise programs to reduce the high rates of HIV transmission between mothers and babies.

Another important consideration was the “local” context with regards to feasibility. Respondents explained this by highlighting the peculiarities of the different regions in the country whereby in one context the issue might be physical access while in the other it might be lack of education:

*“…And then of course take into consideration the context, the different aspects, yes you are one country but one size does not fit it all so take into consideration the different context you know where now when it comes to implementation what do you need to do when you go to Kalangala which is a much more hard to reach district compared to Kampala. What do you need to do when you go to Karamoja where maybe the level of education is much lower?” (N_2).*

Resource availability affects both the practice of setting priorities and the implementation of those priorities. This may be one of the reasons why at the national level, alignment with international declarations and agreements was a consideration, since this secures DAP support.

*“…We do participate in both committees, national policy committees, to kind of understand better the direction of the National Development Plan and looking at the health sector issues in the country based on the studies that have been done and all the evidence that there is in terms of the global indicators whether Uganda is on course to meeting the MDGs that are set for health and if not what the constraints are and if they’re resource constraints in terms of money then we discuss who has which comparative advantage to support which aspect of you know the challenges that government has presented in the NDP…” (DAP_4).*

Other considerations such as political interests were mentioned by a couple of respondents. For example, political considerations are thought to have influenced the PEPFAR’s funding of abstinence strategies, while the President of Uganda was cited to be dissatisfied with circumcision interventions. All other actors: the district, NGOs and DAPs, reported that their programming reflects national priorities.

#### Use of evidence

According to the respondents, the identification of priorities relied heavily on epidemiological evidence presented in the situational analysis, beneficiary assessment data, and the state of the epidemic during the various strategic periods. AIDs surveillance information from the sentinel sites is also relevant to HIV/AIDs prioritization.

#### Reflection of public values

Direct participation is one of the strategies through which public values can be considered. The documents revealed that there are efforts to involve the public in the actual decision making process through consultations, with both political and technical district actors. These consultations were meant to provide public views/input in the development of the national strategy. Further participation of the public is ensured through the annual district partnership meetings, which aim to broaden participation, knowledge and information sharing about HIV/AIDS [22].

#### Publicity of priorities and criteria

Respondents reported on the different strategies used to publicize the priority setting decisions and their rationales. First, all the government documents are available online; second, a number of government priorities were discussed in the media. For example, the government’s implementation of new anti-retroviral treatment policies [38, 39], and the National Prevention Strategy (including prevention of mother to child transmission and male circumcision) [40–44]; the introduction of a new stigma index survey [45], mandatory testing [46], Abstinence, Be faithful and use condom (ABC) strategies, post-exposure prophylaxis, and the integration of HIV/AIDS and sexual reproductive health were also reported [47]. There was also discussion of the controversial HIV prevention bill with some authors supporting it and others noting the potential negative repercussions [48, 49]. It is important to note that most of these are in the print media and mostly in English language. Furthermore, while the priorities were publicized, the rationales for the decisions were not consistently provided.

#### Functional mechanisms for appealing the decisions

No appeals mechanisms were reported. However, there were several complaints against the decisions in the media, for example, grievances about prevention strategies such as the prioritization of male circumcision [37] and the government’s rejection of pre-exposure prophylaxis [50].

#### Functional mechanisms for enforcement

Respondents reported no mechanisms for ensuring that the process was fair and that consistent.

#### Efficiency of the priority setting process

While the prioritization process seemed efficient with the plans being completed and funded within the proposed period, inefficiencies were noted in relationship to implementation of the priorities. In particular, these inefficiencies were attributed to slow bureaucratic and systematic processes. This led to incongruences between the timelines of procuring funds, disbursing secured funds, and implementing prioritized activities.

*“…Yea one of the major challenges that the organization faced is late release of funds. Sometimes funds are released late and then you reach at the time you were supposed to have implemented these activities you know you are…The time lag between release of funds and implementation…”(DAP_2).*

Media reports also alluded to this by reporting on delays in releasing and allocating funds for procuring anti-retro viral drugs, describing stock-outs that resulted from under-quantification by health workers and delays in the release of funds from donors [51, 52].

### Implementation

#### Allocation of resources according to priorities

According to the National AIDS Spending Assessment which captured HIV/AIDS expenditure in 2008/09 and 2009/10, and the first National strategic plan resource estimates, the total HIV expenditure was higher than the planned resource needs [18]. However, the reports also concur with the respondents’ observation in that while the plan was to allocate more resources to prevention, more resources were actually allocated to care and treatment. Moreover, almost 90% of the prevention budget was funded from external sources. However, some of the respondents decried the negative impact that donor funds might have on the implementation of national priorities.

*“…Really donors have taken over this whole sector…they came with their priorities; for HIV they are forcing only on biomedicals, circumcision, but I said you people…this HIV if you don’t touch on people’s morals. You may circumcise people, you may give treatment but if they are not changing their morals and work on prevention they’re wasting their time…they didn’t listen, had their money, they had their way…” (DAP_20).*

#### Decreased resource wastage

An indicator of resource wastage is drug stock-outs and expired drugs. While Uganda has been receiving funding to purchase anti-retro viral drugs ensure that all the people living with HIV/AIDS have access, expiry of the life-saving drugs has been reported in the recent past. The media reported instances whereby almost 54% of sampled health units had expired ARVs, and that $550,000 US dollar worth of HIV drugs had expired in both the clinics and at the national medical stores [52–54].

#### Improved internal and external accountability/reduction of corruption

Accountability, or the ability to monitor and evaluate spending and performance, is a criterion that often determines whether a certain program is likely to be supported. However, the media reported two cases of perceived corruption – the first reported on the alleged misuse of Global Fund grants by government officials in 2005–2006, and the second was on allegations of corruption in the Office of the Prime Minister in 2012 [55–57]. This could be, in part, a consequence of the off-budget funding. As explained by the study respondents.

While the study respondents recognized the need for accountability and the existence of accountability structures, they highlighted the accountability challenges related to HIV/AIDS programming where the bulk of funding is often off budget ---making it difficult for the ministry of health to hold the grantees accountable. As explained by a respondent;

*“…Much of the money, like for HIV/AIDS, for malaria, even reproductive health services, a lot of that money is off budget…it’s controlled by donors, it’s controlled by NGOs, civil society organizations…it’s not easy to extract expenditure on these diseases…. it’s very difficult unless you go to malaria control programme to see how much money is being spent, and HIV/AIDS control programme like that...” (N_1).*

#### Increased stakeholder understanding, satisfaction, and compliance with the PS process

While there were questions with regards to the funding of the different interventions, stakeholders seemed generally satisfied with the ability of HIV/AIDS programs to attract external funding. There were also positive views regarding specific prevention priorities such as male circumcision, the Option B+ program, and other preventive initiatives, which led to significant decreases in new infections [58]. However, there was a feeling that DAPs tended to be promote their own priorities.

#### Decreased dissentions

Many interview respondents relayed dissatisfaction regarding priorities. In particular, the government and donors were described to prioritize treatment while inadequately financing prevention initiatives. These conflicts created difficulty for NGOs and other organizations who were obligated to follow MOH priorities at a time when donors were looking to fund other programs.

*“…there are slightly different priorities that the donors want to fund which might not necessarily be within our docket…We’ve been into HIV Testing and Counsel for a very long time. Circumcision for prevention of HIV came on board about 2-3 years ago and this was, at first, a donor driven activity driven by US, USAID, PEPFAR funding…”(NGO_2).*

The media review revealed a trend in the kinds of dissensions. Earlier dissensions focused on the lack of attention given to Uganda’s HIV/AIDS problem, despite its detrimental impact on the economy [59]. These argued the public and civil society groups to “force” the politicians to prioritize it, with headlines such as: “HIV is a priority that we expect all candidates to address”; “We Are Saying No Drugs, No Votes” [60]. Latter dissensions reiterated the interview findings in part; for example, calling attention on government’s ignoring existing effective prevention strategies; especially the rejection of the pre-exposure prophylaxis strategy in spite of its effectiveness [46, 50]. There were also dissensions in the media about the effectiveness of male circumcision as a preventative strategy [37, 61]. The most recent dissensions were about the implications of the HIV/AIDS Prevention and Control Bill, passed in 2014, which calls for mandatory testing, mandatory disclosure of HIV status, and the criminalization of the intentional spread of the disease [38, 62].

### Impact and outcomes

According to the evaluation framework, successful priority setting should have a positive impact by contributing to the strengthening of the priority setting institution, facilitating positive change in health policy and practice, contributing to the health system achieving its goals, improving financial and political accountability, increasing investments in the health sector and strengthening of the health care system.

### Strengthening of the priority setting institution

The respondents and documents identified the priority setting institutions for HIV as the ministry of health, the National AIDS control program and the Uganda AIDS commission. Respondents reported that before our study the capacity of the HIV specific institutions (as opposed to the ministry of health) had been strengthened through training and increased availability of resources. Hence, it is not possible for us to verify the degree to which the prioritization process contributed to the capacity strengthening.

### Impact on the priority setting institutional goals and objectives

The goals of both the Aids Control Program and the Uganda Aids Commission, as articulated in their policy documents, are to contribute to HIV/AIDS control. The reviewed documents, and respondents confirmed that this goal has been achieved- in the following aspects; they have increased HIV awareness, access to HIV treatment and stabilized HIV prevalence. They have also increased access to HIV counselling and testing, 95% of pregnant mothers were accessing ARV drugs and a reduction in babies with HIV, increased programming and medical circumcision and an increased ART enrollment, increased knowledge of HIV/AIDS and reduced HIV/AIDS related mortality (see Table 5).

**Table 5:** Trends in HIV/AIDs indicators between 2011 and 2015 [23, 24, 34]

Indicator	2011^a^ M/F%	2012^a^ M/F%	2013^a^ M/F%	2014 M/F%	2015^b^ M/F%
Pregnant women on PMTCT	49	87	82	> 95	97%
Knowledge of HIV	39.3/38	36.9/ 29.6	40.3/ 33.1	42.3/35.7	–
Male circumcision	26.4	31.4	35.8	40	–
- > PLHIV receiving ART	25%^b^	33%^b^	43%^b^	53%^b^	57%
AIDS-related deaths	–	–	63,000	31,000^b^	28,000
New infections (adults)	162,294	150,000	140,000	95,000	79,777
New infections (children)	31,000^a^	–	12,000^b^	52,000^b^	3500

^a^[23, 24]

^b^[34]

### Impact on health policy and practice

While it may not be directly attributed to the prioritization process, some policy changes occurred during the study period. Notably, male circumcision was established as one of the priority preventative strategies.

### Achievement of health system goals

#### Improvement of population health

According to the evaluation framework, successful prioritization should in the long run, contribute to improving population health, ensuring fairness in financial contribution, and responding to the public’s expectations. As noted above, the program has improved the quality of life of people living with HIV. Since HIV is an infectious disease, the treatment which is reducing the viral load, the counselling as well as the other preventative methods inherently limit the spread of the virus, hence improving population health.

#### Fairness in financial contribution

Public funding for HIV increased remarkably between 2007/8 and 2011/2012 (from $14 m to $53 m. However, still most of the funding came from external sources, with the out-of-pocket sources estimated at 21%, meaning that households and individuals were contributing greater than the total public contribution [23]. It is not surprising that the most vulnerable may have been most affected, as evidenced by a newspaper report which discusses inequitable coverage of prevention of mother to child transmission, whereby the poorest mothers are unable to access care [63].

#### Responding to the public’s expectations

The interviews and document review did not reveal specific ways through which the priorities enlist and respond to the expectations of the public. Although some of the decisions made during the study period such as the lukewarm response to male circumcision and the Homosexuality bill were allegedly a reflection of what the public valued.

#### Improved financial and political accountability

As reported by both interviewees and media reports, the larger financial and political context of the country impacted funding for HIV during the period under study. There was a deliberate reduction in funding from donors due to issues with poor financial accountability in 2006 and 2012; as discussed above.

#### Increased investment in the health sector and strengthening of the health care system

In 2009–2010, total spending, including private, public, and external sources, for HIV was USD $579.7 million, and $454.2 million respectively; this excluded out-of-pocket household expenses [23, 24]. Both the government and donors have increased spending on HIV/AIDS and the health sector as a whole in the past decade. In 2014–2015, the government of Uganda’s budget for HIV was USD $69.9 million [4].

Respondents discussed funding gaps (in spite of the relatively high funding), coupled with inefficiencies in mobilisation and use of resources. There were also concerns about sustainability and predictability of donor spending, and the impact of the macro-economic landscape on HIV funding in Uganda. Furthermore, a shift of the national priorities to physical infrastructure and wealth creation programs was feared to have hampered the protection of the HIV budget.

Moreover, although the HIV/AIDS program receives substantial funding compared to the other programs; this, as discussed by the respondents, does not translate into increased funding for the health sector as a whole since this funding is often earmarked for HIV. Indeed, this vertical funding is reported to have created challenges and undermined the strengthening of the health system.

## Discussion

To the best of our knowledge, this is one of the few studies to report on a systematic description and comprehensive evaluation of priority setting for HIV. The framework used provided a consistent approach to the evaluation. It enabled us to evaluate most of the critical dimensions and parameters of successful prioritization, beyond the typical evaluations which focus on cost effectiveness and impact on morbidity and mortality.

The study highlighted the close linkages between the priority setting context, the pre-requisites, the process and the implementation and eventual success of the prioritization process; although these are presented as separate domains. Two particular contextual factors namely; the global economic downturn and the political and legal decisions made during the study period seem to have impacted the availability of resources and eventual implementation. While this may have not had an impact in other contexts, the dependency on government on donor funding make it difficult for them to successfully run any program without their support [64]. Furthermore, DAPs, by virtue of their funding the HIV programs, have a strong leverage and do influence priority setting and implementation. This case illustrated the tensions that can exist when the DAPs have priorities that differ from national priorities and the resulting dissensions. This finding is contrary to the literature on stakeholder participation which proposes that stakeholder engagement should increase acceptability and compliance with the set priorities [14]. In this case, while HIV prioritization seemed highly participatory, notably DAPs were thought to champion their own agendas, in spite of the national priorities.

The prioritization process was successful in several aspects; as discussed above it is very participatory, it is based on available evidence, explicit criteria, some of the decisions and criteria are publicized. This could, in part be explained by the presence of legitimate and strong priority setting institutions such as the UAC, which have been politically endorsed and supported [34]. However, while there were criteria used and publicized, not all the criteria is deemed relevant in the literature. Criteria such as politics, although relevant, has been disputed in the literature [65]. Furthermore, while the BOD/CEA approach is used at the health system level in prioritizing HIV; it was unclear if a specific priority setting approach is used when identifying priorities within HIV. An explicit approach would ensure that the decisions are made consistently and are acceptable to all stakeholders. It could potentially contribute to increasing buy in from the different stakeholders, if the approach is perceived as credible [14].

The findings that there lacked explicit mechanisms for appealing/ revising the decisions and enforcement, are consistent with the literature within this and similar contexts [66]. However, the structures within the HIV programming provide an opportunity for testing cross accountability mechanisms whereby the ministry of health keeps the Uganda AIDS commission accountable and vice versa. Furthermore, the two main institutions could consider forming a committee to handle appeals, which would stem the complaints being aired in the media.

The results revealed several critical issues with the implementation of the priorities, first, the budget allocation is inadequate, second, the available resources are not always allocated according to the priorities, third, there were reported cases of resource wastage—as demonstrated by the expiring of drugs. While the latter is an issue of procurement and demand/ supply chain; the former two reflect on the prioritization process. Ideally, priorities are identified in order to ensure that resources are appropriately allocated; hence, one would assume that the priorities should match the resources or vice versa. Unfortunately, it is these cases that introduce “behind closed door” secondary prioritization and resource allocation processes which may not be in line with the participatory processes, and may be perceived as illegitimate [14].

As proposed in the framework, lack of accountability and corruption leads to reduced trust and subsequent investments. This is illustrated in this case study whereby the two instances of corruption led to marked reduction in DAP funding for HIV; with one organization suspending the funding. This limited accountability has been in part blamed on the multiple parallel mechanisms that are introduced with these vertical programs. Often these function independent of the formal structures and may not be held at the same levels of accountability [14, 67]. Streamlining and integrating accountability mechanisms within the priority setting institution as well as the ministry of health would strengthen overall accountability [14, 16, 17].

While the priority setting institutions in the case of HIV, seem to have been strengthened; the degree to which this contributed to the overall strengthening of the health system is questionable. As far as HIV/AIDS as a disease program goes, there have been gains and impact on health policy and practice, population health (see Table 3). However, prioritizing HIV has also had some detrimental impact on the health system due to its verticalization. These findings are not unique and have been documented elsewhere [67]. While vertical planning was essential at the onset of the epidemic, it may be time to rethink the strategy and integrate the services so as to benefit the health sector as a whole, especially in view of the reduction in external funding.

## Study limitations

Due to the study design, we were unable to evaluate some parameters such as stakeholder satisfaction, which would necessitate exit interviews; and the degree of participation of the different stakeholders; which would require direct observation at prioritization meetings; public satisfaction and understanding, which would require public surveys. Furthermore, we report findings that are reported (through interviews and document review); which may not necessarily reflect actual practice. This was mitigated through the triangulation of the sources of information. While there were marked gains in health outcomes (Table 5), given the potential confounding health systems factors, we are unable to attribute these findings entirely to successful prioritization. However, a successful priority setting, and appropriate resource allocation arguably contribute to positive health outcomes, as proposed by the evaluation framework used in this paper.

## Conclusion

We have described and evaluated national level priority setting for HIV in Uganda. The prioritization process was successful in several areas namely; there was a legitimate institution with the capacity to set priorities, the process was participatory and based on credible criteria and evidence, the priorities and dissensions were publicized in the media, some of the priorities were implemented with positive impact on the priority setting institution and health system goals and objectives.

Areas of improvement include: increased accountability, consistent use of an explicit priority setting approach, ensuring that the priorities match the resource envelope and buy in from all stakeholders, especially the DAPs, instituting appeals and enforcement mechanisms to ensure accountability.

The evaluation framework was robust enough to provide insight into the prioritization for HIV in Uganda; beyond cost- effectiveness and mortality and morbidity; demonstrating that program evaluation should include non- technical aspects –such as politics, cultural context, which may impact its success. Most of the parameters were easily assessed; however, due to the study design, parameters that required real time observation were not assessed due to the timing of the study. Future study design should focus on integration of evaluation into the prioritization process so as to ensure that all parameters are assessed.

## Additional file

Additional file 1: Interview Guide. Interview Guide for HIV Policy Makers. (DOC 40 kb)

## Abbreviations

ABC: Abstinence, Be faithful and use condoms
BOD/CEA: Burden of Disease and cost- effectiveness analysis
DAPs: Development assistance partners
eMTCT: elimination of Mother To Child Transmission
LICs: Low income countries
MDGs: Millennium Development Goals
MOH: Ministry of Health
MOV: Means of Verification
OVI: Objectively verifiable indicators
PS: Priority setting
TASO: The AIDS Support Organization
UAC: Uganda AIDS Commission
USD: United States Dollars
